# Seed Removal Increased by Scramble Competition with an Invasive Species

**DOI:** 10.1371/journal.pone.0143927

**Published:** 2015-12-09

**Authors:** Rebecca L. Minor, John L. Koprowski

**Affiliations:** Wildlife and Fisheries Science, School of Natural Resources and the Environment, The University of Arizona, Tucson, Arizona, United States of America; Shandong University, CHINA

## Abstract

Competition for seeds has a major influence on the evolution of granivores and the plants on which they rely. The complexity of interactions and coevolutionary relationships vary across forest types. The introduction of non-native granivores has considerable potential to alter seed dispersal dynamics. Non-native species are a major cause of endangerment for native species, but the mechanisms are often unclear. As biological invasions continue to rise, it is important to understand mechanisms to build up strategies to mitigate the threat. Our field experiment quantified the impact of introduced Abert’s squirrels (*Sciurus aberti*) on rates of seed removal within the range of critically endangered Mount Graham red squirrels (*Tamiasciurus hudsonicus grahamensis*), which consumes similar foods. In the presence of invasive Abert’s squirrels, the time cones were removed was faster than when the invasive was excluded, accounting for a median removal time of cones available to red and Abert’s squirrels that is 32.8% less than that of cones available only to the rare native red squirrels. Moreover, in the presence of Abert’s squirrels, removal rates are higher at great distance from a territorial red squirrel larderhoard and in more open portions of the forest, which suggests differential patterns of seed dispersal. The impact on food availability as a result of cone removal by Abert’s squirrels suggests the potential of food competition as a mechanism of endangerment for the Mount Graham red squirrel. Furthermore, the magnitude and differential spatial patterns of seed removal suggest that non-native granivores may have impacts on forest regeneration and structure.

## Introduction

Population dynamics of many species of forest rodents, such as tree squirrels, fluctuate with food availability [1]; food addition experiments typically result in increased population density, increased body weights, and early breeding [2]. Competition among the seed eating guild of birds, mammals and insects within forests is often extreme [3, 4] and the long associations with considerable fitness relationships between granivores and trees have led to coevolutionary relationships [5, 6, 7]. Among seed-eating granivorous rodents, specialized dentition [8], hoarding strategies [9, 10], and food handling behavior [11, 12] are likely adaptations to competiton for seeds [5, 13]. However, the introduction of non-native species can result in fundamental changes to long-evolved relationships yet are poorly understood [14, 15].

Interactions between native and non-native species have led to decline of native species in many ecosystems [16, 17]. Worldwide, non-native species rank among the top 5 causes of endangerment of native species [18, 19]; however, mechanisms by which non-native species cause endangerment of native species are often unclear. Disease transmission, predation, hybridization and competition are among the common mechanisms that have been implicated [17, 20, 21].

Systems with limited resources into which non-native species are introduced provide an excellent opportunity to investigate endangerment of native populations by competitive interactions. Tree squirrels are small mammals for which fitness is closely tied to availability of food resources [1, 22, 23] and introduction of competitors could affect food availability. For example, recent declines in Eurasian red squirrels (*Sciurus vulgaris*) in the British Isles and Italy are attributed in part to a lack of resource partitioning with introduced eastern gray squirrels (*Sciurus carolinensis*; [16]). Similarly, concerns were expressed about introduction of Abert’s squirrel (*Sciurus aberti*) into isolated mountain ranges in the southwestern United States that harbored endemic Arizona gray squirrels (*S*. *arizonensis*: [24]) and federally endangered Mt. Graham red squirrels, a population which is in danger of extinction (*Tamiasciurus hudsonicus grahamensis*: [25]). Use of mixed conifer and spruce-fir forests by Abert’s squirrels creates significant spatial and dietary overlap with the endangered red squirrels [25, 26, 27] for the most common food resources consumed by red squirrels [26]. Computer modeling suggests that this use of cone crops by Abert’s squirrels within red squirrel habitat, and their likely competition with red squirrels for food, may negatively impact red squirrel abundance [28]. Abert’s squirrels are known to consume up to 74% of the annual cone crop in their native range [29]. The relatively recent introduction of the Abert’s squirrel to the Pinaleño Mountains may have increased competition with the red squirrel for a limited food source.

Interspecific competition is often difficult to observe in the field [21]. Investigations of naturally coexisting species with similar requirements often reveal a lack of direct competition due to niche partitioning and other avoidance strategies [30, 31]. Experimental manipulations in the field, such as removal and exclusion studies, present elegant methods to discern the presence of interspecific competition [32, 33]. Our study seeks to address questions about how seed removal is influenced by the presence of an introduced species through investigating cone removal rates using controlled experimentation. We hypothesized that the introduction of Abert’s squirrels in the Pinaleño Mountains may be a factor contributing to the endangerment of the Mount Graham red squirrel, as increased cone removal may reduce food availability for red squirrels in this system where food is already a limiting resource, therefore potentially acting as a mechanism of endangerment. As a test, we quantified the extent to which rates of cone removal were increased in the presence of Abert’s squirrels in this isolated range. In addition, red squirrel defense of central middens [34] led us to investigate the relationship between red squirrel middens, habitat characteristics and cone removal rates. Herein, we used a novel exclusion experiment to quantify cone removal rates in the presence and absence of Abert’s squirrels that enabled us to assess the considerable influence of this invasive species on seed loss.

## Methods

Field efforts were conducted under permits from the United States Department of Agriculture Forest Service, Arizona Game and Fish Department, and United States Fish and Wildlife Service, and approved by the University of Arizona Institutional Animal Care and Use Committee (IACUC; Protocol #08–024).

### Study organisms

Endemic red squirrels are small bodied tree squirrels (mean 231 g, range 190–290 g; [35]) that maintain larderhoards, or middens, which facilitate food storage [36]. Individuals defend a small home range including a central midden (0.301 ha, range 0.285–0.317 ha; [37]), with agonistic inter- and intraspecific interactions [27]. Red squirrels inhabit spruce-fir and mixed conifer forests above 2 133 m. The primary food sources for red squirrels are conifer cones, fungi, and sap [28, 38]. Red squirrels are associated with dense forest characterized by greater canopy cover (90%, range 51–100%), total basal area (73.9 m^2^/ha, range 30.1–165.8 m^2^/ha), and volume of logs (331.3 m^2^/ha, range 0.0–1,295.5 m^2^/ha), compared to random sites [25, 36, 37, 39, 40]. Increased canopy cover promotes protection from avian predation and cool microhabitats, which increase the functionality of the midden for food preservation and logs provide easy movement across the forest floor [25, 36, 37, 39, 40].

Introduced Abert’s squirrels are large bodied (613 g, range 455–824 g; [41]) and occupy home ranges that are 35 times larger than those of red squirrels (10.55 ha, range 6.3–20.3 ha; [42]) and lack a central larderhoard. Abert’s squirrels in the Pinaleño Mountains prefer sites with large live trees (> 40 cm DBH), high tree species diversity, and open, fire damaged forest [25, 43, 44]. Abert’s squirrels may use large trees that are more suitable for the construction of leaf nests, and open forests because they do not cache cones and thus do not need the protection of shade to keep caches cool [25 43, 44]. Douglas-fir (*Pseudotsuga menziesii*) and Engelmann spruce (*Picea engelmannii*) cones are commonly eaten foods for both squirrel species in the Pinaleño Mountains [26, 28].

### Study area

We conducted our field experiment during two consecutive years in autumn, the season when conifer cones are collected and cached by squirrels in the Pinaleño Mountains of southeastern Arizona, USA. This range supports the entire population of Mount Graham red squirrels [45], hereafter referred to as red squirrel. Abert’s squirrels were introduced to the Pinaleño Mountains in 1941 by the Arizona Game and Fish Department to increase recreational hunting opportunities [46]. The population of red squirrels in the study area is part of a long-term monitoring project [47]. The Pinaleño Mountains are typical of other conifer forests, where trees reproduce in mast cycles, producing more cones some years than others [42]. Experimental locations were randomly selected throughout areas occupied by Abert’s and red squirrels in mixed-conifer forest, and were largely unaffected by recent bark beetle outbreaks (1998; [40]) and catastrophic fire (2004; [48]).

### Cone removal rates

We collected green Douglas-fir and Engelmann spruce cones outside the study area but within the Pinaleño Mountains. Green cones are those that contain ripened seeds still folded within the protective scales of the cone. Cones at this stage provide nutrition to seed predators with strong jaws, such as tree squirrels, that are able tear open the scales and access the seeds within. Both red and Abert’s squirrels consume and transport green cones during early autumn, and red squirrels cache cones for the coming winter [9, 40, 42]. Green cones collected at the beginning of each field season were stored during the study period at 5°C to prevent mold and opening prior to use in field experiments.

Experimental plots were established at random locations throughout the study area in the autumn. We placed cones on a 4 m × 4 m grid at 1 m spacing (16 cones / plot). Engelmann spruce and Douglas-fir cones were randomly selected for inclusion such that 8 cones of each species were placed on each plot. We observed experimental plots directly with binoculars and remotely through infrared trail monitoring systems and cameras (Trailmaster TM550 with TM 35–1 Camera kit, Goodson and Associates, Lenexa, KS). Animals foraging on experimental plots were identified directly by personal observation and remote camera observation of squirrels, and indirectly by observations of feeding sign that can be distinguished to species level because of the 3-fold difference in size between the red and Abert’s squirrels’ bite marks [35, 42]. In three replicate trials, cones were available for an average of 6 days, after which we revisited plots to record removed cones.

**Fig 1:**
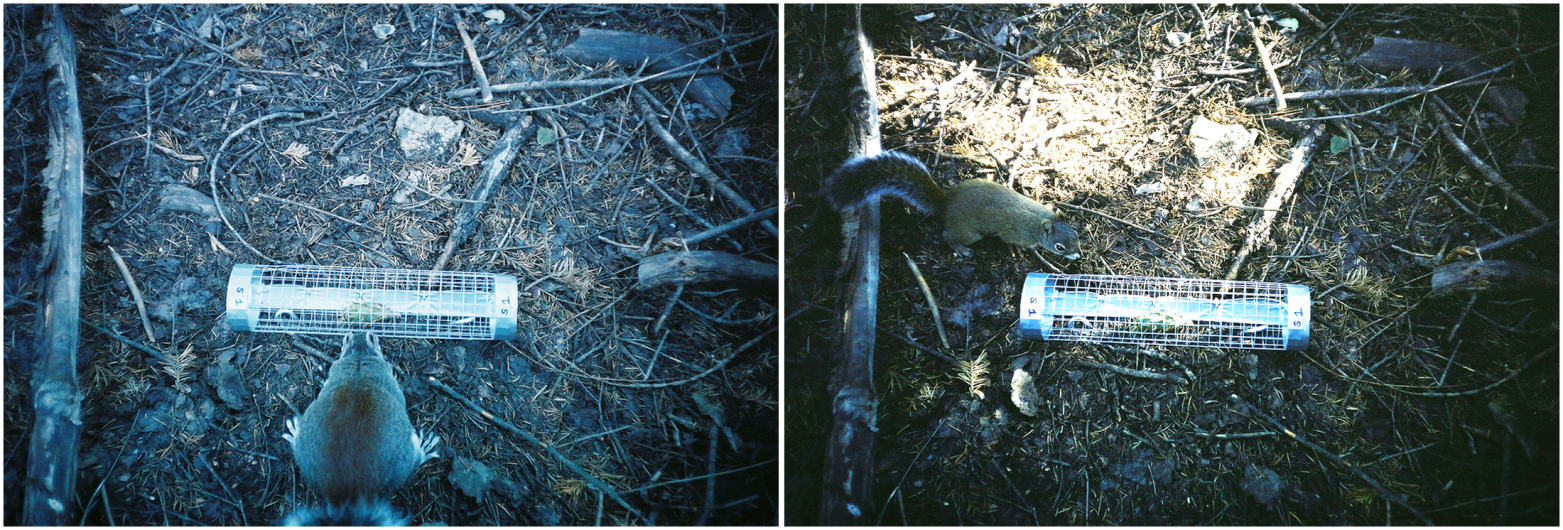
Activity at an Abert’s squirrel (*Sciurus aberti*) exclusion cone tube, Pinaleño Mountains, Autumn 2008. Abert’s squirrels (*Sciurus aberti*, left) were excluded from access to cones in this treatment, whereas Mount Graham red squirrels (*Tamiasciurus hudsonicus grahamensis*, right) were able to remove cones.

We also conducted an exclusion experiment with two treatments to assess rates of cone removal in the presence and absence of the population of introduced Abert’s squirrels: exclusion cone tubes that allow access to only the smaller red squirrel (diameter = 8 cm, n = 15), and control cone tubes that allow access to both species (diameter = 12 cm, n = 15; Fig 1). We constructed these cone tubes from galvanized 2.54 cm mesh hardware cloth closed with vinyl zip ties and covered sharp edges with duct tape. We randomly placed 30 cone tubes baited with 1 cone across the study area inhabited by Abert’s and red squirrels and anchored the tubes with 12 cm long metal stakes. We checked the 30 cone tubes every 4–7 days and recorded presence or absence of the cone. We replaced absent cones, cones that were open with seeds exposed, and cones that were exposed for more than 20 days (early in the study) and 14 days (late in the study) without being removed by a seed predator. With each new green cone, we relocated cone tubes at a randomly selected bearing ≤ 20 m from the initial placement point to maintain the independence of each cone’s rate of removal. The resulting encounter histories allowed us to compare removal rates of cones available to only red squirrels (exclusion cone tubes), and cones available to both populations of tree squirrels in the study area, Abert’s and red squirrels (control cone tubes). In addition, 10 cone tubes (5 of each treatment) were monitored by remote cameras (Trailmaster TM550 with TM 35–1 Camera kit, Goodson and Associates, Lenexa, KS) to confirm the ability of excluders to effectively exclude Abert’s squirrels, and the ability of both squirrel species to remove cones from cone tubes.

### Measurements of local features

We measured forest-stand structure and vegetation characteristics in 10 m radius plots [40] centered about the initial placement point of the 30 cone tubes in order to investigate the influence of forest structure on cone removal rates. We recorded average canopy cover determined by densiometer readings at 0, 5, and 10 m in the 4 cardinal directions as an indicator of forest openness.

Red squirrel middens across the study area are visited quarterly to assess level of occupancy at these larderhoards, resulting in an estimate of population abundance [49]. Thus, to investigate the relationship between red squirrel middens and cone removal rates, we used GIS software to plot locations of all middens occupied in September and December and the locations of all cones placed on the landscape from September to December (ArcMap, ESRI, Redlands, CA). We used a distance and azimuth tool to spatially analyze the distance between each experimental cone and the nearest occupied red squirrel midden.

### Statistical analyses

We made several comparisons to examine the extent to which cone removal rates increased in the presence of Abert’s squirrels in the Pinaleño Mountains. All data on cone removal were interval censored as the removal occurred during the time interval between the date the cone was last observed, and the next observation at which the cone was absent. Right censoring was applied to cones that opened and were replaced by researchers during the study or were still present at the end of the study, indicating a conservative survival estimate that is at least the number of days the cone was under observation. The oldest cone to be removed by a squirrel (therefore not right censored) was found absent after 17 days of observation. Thus, the last interval to include an uncensored value ended on day 17, leading to the decision to truncate these data at day 17 and represent them by a Weibull distribution. We compared, with a parametric survival model, removal rates of cones available to Abert’s and red squirrels in the control cone tubes and on experimental plots to assess any effect of the cone tube apparatus (JMP 8, SAS Institute, Cary, NC). All analyses of cone removal from cone tubes include only Douglas-fir cones due to low sample sizes of Engelmann spruce cones from cone tubes. Finally, we built a parametric survival model with a Weibull distribution that included the variables treatment, date of entry to the study, and their interaction to investigate the effect of cone tube treatment; that is, addition or exclusion of Abert’s squirrels. The date on which a cone entered the study was included in the model to address time-dependent variation over the 106 day study period from early September to mid-December. Because no interaction was detected, we interpreted a reduced model that did not include the interaction.

We examined the influence of proximity to an active red squirrel midden on cone removal rates with a parametric model of red squirrel cone removal and of the removal rates of cones available to both squirrel species. These models also followed a Weibull distribution. We included date of entry to the study, distance (m) to nearest red squirrel midden, and the 2-way interaction in the models.

A parametric survival model with canopy cover, date of entry into the study, and the 2-way interaction investigated the relationship between the probability of cone removal by red squirrels and forest density. Analyzing removal rates of cones available to both red and Abert’s squirrels with the same variables explored the relationship of probability of cone removal in the presence of red and Abert’s squirrels with more dense forest. These models included one randomly chosen cone at each of the 30 initial placement locations to represent removal rates in the area at which canopy cover was measured.

## Results

### The impact of Abert’s squirrels on cone removal rates

Abert’s and red squirrels removed cones from experimental plots, supporting previous observations that both species do forage on the ground during autumn [9, 27, 42]. Remote camera photographs and direct observations of animals and feeding sign documented use of experimental plots by Abert’s and red squirrels. Of 240 cones placed on plots, 60.8% (n = 146) were removed. Among those removed, 61% were Douglas-fir and 39% were Engelmann spruce (Χ^2^ = 17.94, *df* = 1, *p* ≤ 0.0001).

**Fig 2:**
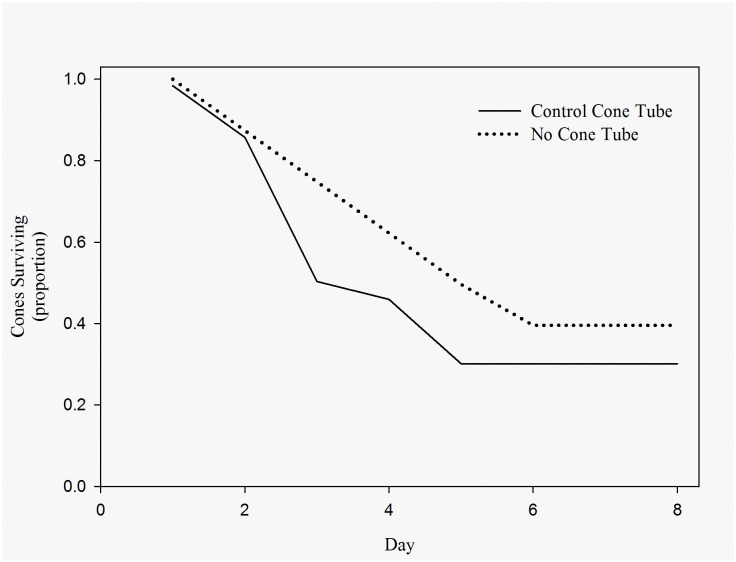
Comparison between experimental plots and cone tubes. The removal rates of cones placed within a control cone tube (solid line) or on experimental plots not in a cone tube (no cone tube: dotted line) and available to both Mount Graham red squirrels (*Tamiasciurus hudsonicus grahamensis*) and Abert’s squirrels (*Sciurus aberti*) in the Pinaleño Mountains, Arizona during our field experiments.

Cone placement within a cone tube did not affect rate of removal. Removal rates of cones available to Abert’s and red squirrels on plots were not different from removal rates of cones placed on the study area in control cone tubes, also available to both Abert’s and red squirrels (Χ^2^ = 2.41, *df* = 1, *p* = 0.121, parametric survival; Fig 2). Both Abert’s and red squirrels removed cones from control cone tubes, as documented by direct observation and remote camera photographs. However, Abert’s squirrels did not remove cones from exclusion cone tubes, whereas red squirrels entered the small tubes and removed cones (Fig 1).

**Fig 3:**
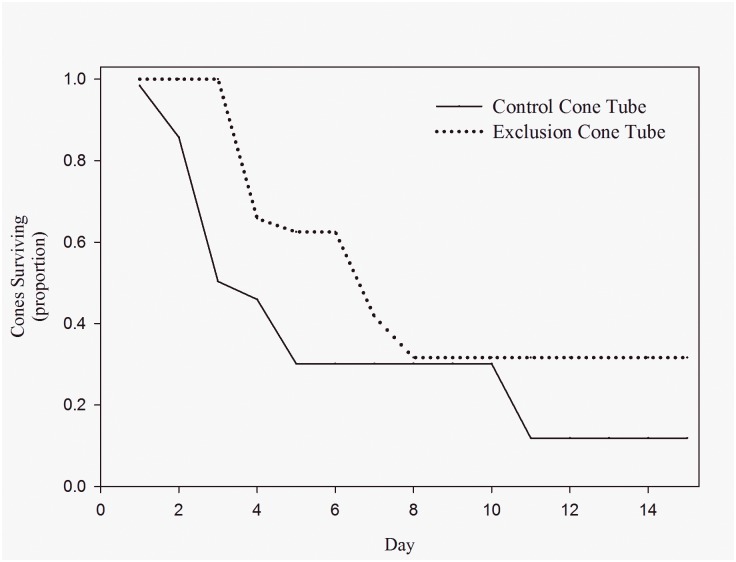
Exclusion experiment cone removal rates. Removal rates of cones available to both endangered Mount Graham red squirrels (*Tamiasciurus hudsonicus grahamensis*) and introduced Abert’s squirrels (*Sciurus aberti*) (control cone tube: solid line) and cones from which Abert’s squirrels are excluded (exclusion cone tube: dotted line) in the Pinaleño Mountains, Arizona.

Making cones accessible to Abert’s squirrels had a large impact on cone removal rates after accounting for the cone’s date of entry into the study. We found a 1.6% increase in time to removal for every 1 day later in the study (Χ^2^ = 14.33, *df* = 1, *p* = 0.0002, reduced model, parametric survival). In the presence of Abert’s squirrels, 50% of cones were removed after only 7.3 days (95% confidence interval from 5.48 to 9.67 days), whereas 50% of cones available only to red squirrels were removed after 12.8 days (95% confidence interval from 7.73 to 21.30 days). Thus, in the presence of Abert’s squirrels, the time until 50% of cones were removed was 5.6 days faster than when Abert’s squirrels were excluded (95% confidence interval from 2.25 to 11.63 days). The median time to removal among cones available to red and Abert’s squirrels was 32.8% less than that of cones available only to red squirrels (Χ^2^ = 4.05, *df* = 1, *p* = 0.04, reduced model, parametric survival; Fig 3). Before any cones were removed from the exclusion cone tubes, a proportion of 0.496 (SE = 0.134) cones available to both red and Abert’s squirrels were already removed from the control cone tubes.

### The impact of season and local environmental features on cone removal

Among cones available to only red squirrels, distance to an active midden and the date of entry into the study interacted such that cones had a higher probability of removal further from middens early in the autumn, and closer to middens later in the autumn (Χ^2^ = 3.98, *df* = 1, *p* = 0.046, full model parametric survival). However, the median time to removal of cones available to both red and Abert’s squirrels decreased 4.6% for every 10 m closer to active middens (Χ^2^ = 11.12, *df* = 1, *p* = 0.0009, reduced model, parametric survival), and increased 1.3% for every 1 day later in the autumn (Χ^2^ = 8.53, *df* = 1, *p* = 0.0035, reduced model, parametric survival).

Among cones available to both Abert’s and red squirrels, the median time to removal decreased 8.03% with every 1% increase in canopy cover (Χ^2^ = 8.48, *df* = 1, *p* = 0.0036, reduced model, parametric survival), and increased 3.5% with every 1 day later in the autumn (Χ^2^ = 8.94, *df* = 1, *p* = 0.0028, reduced model, parametric survival). However, the probability of removal among cones available only to red squirrels was not related to levels of canopy cover (Χ^2^ = 0.32, *df* = 1, *p* = 0.5727, reduced model, parametric survival), although we found a 4.4% increase in the median time to removal for every 1 day later in the autumn (Χ^2^ = 4.05, *df* = 1, *p* = 0.0441, reduced model, parametric survival).

## Discussion

Tree squirrels in general, and *Tamisciurus* in particular, are often thought of as indicator species for forest health [50] with life histories that reflect the seasonal nature of their food sources [51], including reproduction when food is plentiful [35, 52], and the use of cached resources when food becomes scarce [42, 53]. Reliance on annual cycles of food availability is apparent in the higher cone removal rates observed in early autumn in the Pinaleño Mountains. In late autumn and early winter, we observed cone removal rates diminishing as expected with the changing of the seasons as squirrels constrict their home ranges [41, 42] and conifer cones open, allowing seeds to fall to the ground and making them available to all seed predators [54].

Abert’s squirrels have been classified as ponderosa pine obligates, and their native distribution includes ponderosa pine forests throughout the southwestern United States and northwestern Mexico [42]. In the Pinaleño Mountains, Abert’s squirrels are present over a wide range of vegetation types including spruce-fir and mixed-conifer forests [25, 26, 27], yet have been shown to maintain a preference for open forest structure in these novel forest types [25, 43, 44]. In our study of cones available to red and Abert’s squirrels, the positive association between cone removal and canopy cover suggests that Abert’s squirrels are moving into more dense areas to forage in the Pinaleño Mountains. This result suggests that introduced Abert’s squirrels not only inhabit forest types populated by red squirrels, but are removing cones from areas of habitat known to be preferentially selected by red squirrels within these forest types. This finding supports the contention that the introduction of Abert’s squirrels in the Pinaleño Mountains may be a factor contributing to the endangerment of the Mount Graham red squirrel as cone removal by Abert’s squirrels could negatively impact red squirrels [28].

In some systems, invasions are dampened and impacts lessened when natives are capable of biological resistance [21, 55]. Territoriality is one form of biological resistance, effectively diminishing the available habitat for would-be invaders by competition for resources within the territory [56, 57]. Removal of cones available only to red squirrels reveals a relationship between seasonality and proximity to an active midden where red squirrels remove cones further from middens early in autumn, and closer to middens later in autumn. This result suggests that red squirrels forage further away from middens early in the autumn when cones have not yet been cached in the middens, however, later in the autumn when middens are full of cones and need to be protected [58], red squirrels stay closer to these sensitive areas. Although red squirrels chase Abert’s squirrels from occupied middens [27], and kleptoparasitism is rarely observed [59], a negative relationship between distance and removal probability was observed in cones available to both species, showing a higher probability of removal closer to red squirrel middens and suggesting an impact of Abert’s squirrels on patterns of cone removal near middens, areas that are critical to red squirrels.

As on islands and other isolated ranges where inhabitants have been separated from the selection pressures of competition, Mount Graham red squirrels may lack strategies to combat the introduction of Abert’s squirrels [17, 20, 57, 60, 61]. In the ponderosa pine forests of the southwestern United States where both Abert’s and red squirrels are native, the species coexist on mountain ranges by apparent niche partitioning, such that Abert’s occupy the low-elevation ponderosa pine stands and red squirrels dominate the high-elevation spruce-fir forests [42, 48].

Ecologists have the opportunity to observe ecological processes in action as non-natives invade new environments [21, 57]. A superior competitive ability in accessing resources is implicated as the mechanism by which several invading species have successfully become established in their non-native ranges [21, 62, 63]. Notably among tree squirrels, the eastern gray squirrel has had a negative impact on the Eurasian red squirrel as a result of a 70% niche overlap between the two species with no evidence for niche partitioning [16]. A similar phenomenon is observed in the Pinaleño Mountains, where Abert’s squirrels occupy a much broader range of habitats than do the native red squirrels [25]. Abert’s and red squirrels exhibit the potential for food competition as both ate cones placed on experimental plots and in cone tubes in our experiment (Fig 2), and have been reported to eat similar foods in other studies [27, 38].

Abert’s squirrels were introduced 70 years ago and are now well established, occurring in high densities across the Pinaleño Mountains [39]. We believe that the difference in cone removal rates observed in this study is evidence that Abert’s squirrels could be impacting red squirrel access to food resources. In the presence of Abert’s and red squirrels, half of the cones available were removed before any cones available to only red squirrels were removed (Fig 3). Such reductions in cones available to red squirrels may have severe consequences for red squirrel persistence [1, 51]. As model predictions indicate, the importance of cone crop reduction by Abert’s squirrels within red squirrel habitat is a threat with great potential to negatively impact red squirrel abundance [28]. Thus, the introduction of the non-native Abert’s squirrel to the Pinaleño Mountains represents potential competition that may threaten persistence of one of the most critically endangered species in North America [49].

The management techniques available to combat resource competition between exotics and natives require creativity and persistence [64]. Efforts to remove Abert’s squirrels from the Pinaleño Mountains have begun with recreational hunting across all seasons and an unlimited daily take. The success of removal by hunting may be limited and a variety of methods, including targeted trapping and removal efforts, may be necessary [60]. The Pinaleño Mountains boast extremely rugged terrain with steep slopes and difficult access across the range. Because of this, complete removal of the introduced Abert’s squirrel may never be achieved. Further studies are needed to elucidate the feasibility of red and Abert’s squirrel long-term coexistence in the Pinaleño Mountains and address the relative appropriateness of strategies that emphasize either eradication, control or forest management [57, 65].

Seed removal is clearly influenced by invasive Abert’s squirrels with the potential for impact on red squirrel fitness; however, the potential impact on forest dynamics is unknown. Seed loss to non-native granivores is an issue in other sites of introduction [14]. Introduced eastern gray squirrels reduced the fitness of native Eurasian red squirrels through pilferage of cache sites [16] and significant portions of seeds are lost to introduced granivores including wild boar (*Sus scrofa*) in forests of Argentina [66] and murid rodents in timbered areas of New Zealand [67, 68]. The long-term consequences of the altered seed removal patterns are unknown. Abert’s squirrels rarely cache cones and scatterhoad when they do [69]; however, red squirrels are primarily larderhoarders suggesting that spatial patterns of seed dispersal will be modified in addition to abundance of seeds. However, the scant evidence available on introduced seed predators suggest that negative consequences can result from reduced seed dispersal by native seed predators, placement in poor sites for germination, and changing seed shadows [14]. Examination of altered seed dispersal patterns and consequences to forest dynamics will be necessary to study emergent properties of natural systems following biological invasion.

## Supporting Information

SI Dataset Data used in analyses. (XLSX)
